# Alterations in serum and intestinal ACE2 in Inflammatory Bowel Disease and the impact of inflammation

**DOI:** 10.3389/fgstr.2025.1590646

**Published:** 2025-09-10

**Authors:** Fiona Jones, Ciara Egan, Miriam Tosetto, Moritz Strowitzki, Sarah J. Kierans, Catherine Rowan, Margaret Walshe, Elizabeth Ryan, Juliette Sheridan, Garret Cullen, Hugh Mulcahy, Sean Martin, Maura Cotter, Cormac T. Taylor, Glen A. Doherty

**Affiliations:** ^1^ Centre for Colorectal Disease, St. Vincent’s University Hospital, Dublin, Ireland; ^2^ School of Medicine and The Conway Institute, University College Dublin, Dublin, Ireland; ^3^ Department of Biological Sciences, University of Limerick, Limerick, Ireland

**Keywords:** Inflammatory Bowel Disease, angiotensin converting enzyme 2, COVID-19, inflammation, ulcerative colitis

## Abstract

**Background/aims:**

ACE2 is highly expressed in the gut and with known alterations in expression in IBD patients potentially linked to gut inflammation and fibrosis. In addition, little is known about the role of serum soluble ACE2 (sACE2) or its hypothetical role in SARS-CoV-2 binding. We sought to evaluate tissue and serum ACE2 profiles in IBD and healthy controls and evaluate alterations related to disease activity and medical therapy.

**Methods:**

Circulating sACE2 and intestinal tissue ACE2 was evaluated respectively in serum samples and endoscopic biopsies from patients with IBD and healthy controls in addition to murine DSS induced colitis.

**Results:**

91 IBD (UC/n=41; CD n=50) and 55 controls were analyzed. Immunohistochemical ACE2 staining in controls was limited to brush border expression with markedly increased colonic ACE2 expression (and reduced ileal ACE2 expression) in IBD. This was not observed in the mouse model which demonstrated positive ileal ACE2 and negative colonic staining in healthy and DSS mice. Colonic ACE2 staining was further increased in Ulcerative Colitis in inflammation (% staining, 20(5-30) vs. 5(0-6.5), p<0.015) and in IBD patients receiving corticosteroids (% staining, 20(20-40) vs 10(0-20), p<0.052). Steroid use was associated with significantly lower sACE2 with a trend towards reduced sACE2 with biologic exposure.

**Conclusion:**

We observe significant increases in colonic ACE2 expression in IBD, especially with active colitis. Corticosteroids further modify the observed imbalance between tissue and serum ACE2 levels.

## Highlights

- What is already known? ACE2 is highly expressed in the small intestine and is known to be altered in IBD patients with available evidence, although somewhat conflicting, showing increases in colonic and reduced ileal expression in IBD patients. The pathophysiology of altered ACE2 in IBD and its potential role in inflammation and fibrosis in IBD is not known. In addition, despite increasing data on ACE2 luminal expression, little is known about the role of serum soluble ACE2 (sACE2) and its potential role in SARS-CoV-2 binding.
- What is new here? Here we compare ACE2 expression profiles across a model of acute inflammation in the DSS mouse with the chronic inflammation seen in IBD patients. There were no alterations in ACE2 expression in the acute model of inflammation seen in the mouse model. Conversely, we confirm significantly altered ACE2 expression in IBD patients with further alterations in those with active disease. In addition, we demonstrate significantly altered serum sACE2 levels in IBD with the highest levels in male patients with ulcerative colitis and significantly lower levels in those on corticosteroid therapy.
- How can this study help patient care? Our study describes the intestinal alterations in ACE2 in IBD and demonstrates that this may relate to chronic as opposed to acute inflammation. It also sheds important light on the potential alterations in circulatory ACE2 in inflammation in IBD.

## Introduction

ACE2 is a transmembrane glycoprotein well-known for its role in cardiovascular and renal physiology where it cleaves several peptides of the renin-angiotensin system (RAS). The COVID-19 global pandemic has led to increased scrutiny of ACE2 where it’s expression on type II pneumocytes and many other tissues facilitates SARS-CoV2 viral entry into cells (1). The intestine has the highest ACE2 levels in the body however, its role in gastrointestinal health and disease is yet to be fully delineated (2). In addition to its potential role in mediating fibrosis via attenuation of the RAS, ACE2 is involved in the stabilization of amino acid transport and plays a key role in the regulation of intestinal homeostasis, where its deficiency may increase susceptibility to intestinal inflammation (3–5).,Although membrane bound ACE2 is the predominant form in the body, low levels of soluble ACE2 (sACE2) circulate in the blood as a result of cleavage of membrane bound ACE2 by the disintegrin and metalloproteinase, ADAM17 (6). Scant data is available on the role of sACE2 in IBD or alterations that may exist in disease states.

To date huge variations in COVID-19 viral susceptibility have been identified, with the vast majority of patients hospitalized having at least one co-morbidity including older age, metabolic and cardiovascular risk-factors (7). Global data from the SECURE-IBD registry has not identified a signal of increased overall risk of COVID-19 susceptibility or severity in IBD patients but published data demonstrates an increase in COVID-19 severity and death in older patients, male patients, and those taking corticosteroids, with a lower risk in patients taking biologic therapies (8, 9). The reasons for this remain unclear but hypothetically may relate to alterations in ACE2 levels in these patients, with some studies showing anti-TNF therapy led to downregulation of intestinal ACE2 expression (10, 11).

Emerging data on the expression profiles of ACE2, in particular the impact of soluble ACE2, in patients with IBD remains somewhat conflicting. The objectives of this study were therefore to further evaluate the circulating and tissue levels of ACE2 in patients with inflammatory bowel disease, compared to healthy controls and assess the impact of medical therapy and inflammatory disease burden. Additionally, we investigated ACE2 expression in healthy and mouse intestinal tissue and in the dextran sodium sulfate (DSS) model of colitis to explore the role of acute (DSS) vs chronic (human IBD) intestinal inflammation in ACE2 expression.

## Materials and methods

### Animal model- biospecimen acquisition, treatment and ACE2 immunohistochemistry (mouse tissue)

Female C57BL/6 mice aged between 10 and 12 weeks were used in animal experiments. Mice were obtained from and maintained at the Biomedical Facility in the Conway Institute in University College Dublin. Procedures were reviewed and approved by the University College Dublin Animal Research Ethics Committee. Mice in the DSS group were administered 2.5% (wt/vol) DSS (molecular weight: 36,000–50,000; MP Biomedicals) in their drinking water to induce colitis over a period of 6 days. DSS treated mice developed a marked increase in disease activity index (DAI), a composite score based on percentage weight loss, presence of fecal occult blood and stool consistency from day 3 onwards. On day 6, all mice were euthanized by cervical dislocation. After euthanasia, the colon of each mouse was removed, rinsed with PBS and 1cm sections of mouse distal colon +/- ileum were fixed in 10% (vol/vol) formalin before being embedded in paraffin wax.

ACE2 immunohistochemistry was performed on 4µm sections of paraffin-embedded tissue specimens. Deparaffinized slides were heated at 92°C for 10 minutes in antigen retrieval buffer (10 mM sodium citrate, pH 6). Sections were incubated for 10 minutes in permeabilization buffer (0.25% Triton X100, PBS), washed in PBS-T (0.1% Tween‐20, PBS), and incubated in 3% H_2_O_2_ for 10 minutes. Sections were blocked in 10% horse serum in PBS for 1 hour, washed in PBS-T, and blocked with avidin blocking solution (Vector Labs) for 20 minutes (x2). Sections were incubated overnight with ACE2 Ab (1:125, Santa Cruz, SC- 390851). Following primary incubation, slides were developed with Vectastain Universal Elite ABC Kit according to the manufacturer’s instructions, and counterstained with haematoxylin. Images of sections were acquired using an Aperio ScanScope XT at 40× magnification.

### Study population and biospecimen acquisition (human participants)

Participants were included from subjects originally recruited in prospective cohorts of patients with IBD at the Centre for Colorectal Disease biorepository, St. Vincent’s University Hospital, Dublin with the timing of samples relating to prior study protocols. All participants were recruited from 2016-2019. Previous publications relating to these datasets can be found in supplementary materials (12, 13).

Endoscopic tissue (terminal ileum and/or colon) and/or serum samples were collected from patients or controls undergoing colonoscopy as part of routine clinical care or commencing or attending for biologic infusions, with additional control volunteers without IBD from staff at St. Vincent’s University Hospital for serum ACE2. Patients in cohort 1 had a confirmed diagnosis of Crohn’s Disease (CD) and had serum blood samples taken prior to and following anti-TNF administration. Patients in cohort 2 had a confirmed diagnosis of CD with serum samples taken prior to ustekinumab administration. A subset of patients in both CD cohorts had contemporaneous terminal ileal +/- colonic biopsies taken at endoscopy. Patients in cohort 3 had a confirmed diagnosis of Ulcerative Colitis (UC) and had serum samples taken during scheduled appointments for endoscopic assessment. Endoscopic biopsies were obtained using standard forceps from inflamed +/or normal areas of ileum and/or colon where applicable. All patients provided written informed consent to participate in the studies. Clinical and demographic data was collected during patient interview during enrolment in each specific study with further data relating to this follow up study collected retrospectively from our hospital IBD database. Active disease was defined as an endoscopic Mayo scope of ≥1 or evidence of endoscopic evidence of ileal inflammation. The study was reviewed and approved by St. Vincent’s University Hospital Research Ethics Committee.

### Immunohistochemistry (human tissue samples)

Tissue biopsies were fixed overnight in 4% paraformaldehyde (PBS, pH 7.4) and paraffin embedded. 4μm sections were cut, deparaffinized and stained with haematoxylin and eosin (H&E) for assessment of histological activity. ACE2 immunohistochemistry was performed on 4µm sections of paraffin-embedded tissue specimens (Dako EnVision™ kit). Deparaffinized biopsy sections were blocked with hydrogen peroxide, and heated in a pressure cooker for 5 minutes in antigen retrieval buffer (10mM citric acid and sodium citrate buffer, pH 6). Sections were incubated with blocking buffer (Casein, (Vector Lab #SP-5020), washed in PBS-T (0.05% Tween-20, PBS), then incubated with primary antibody (R&D Systems) overnight. Sections were incubated with secondary antibody for 30 minutes (Dako/HRP) followed by diaminobenzidine chromogenic substrate (DAB+, Dako) for 10 minutes prior to washing and counterstaining with haematoxylin. Slides were qualitatively analyzed for localization of specific antibodies by a blinded pathologist.

### Soluble ACE2 measurement by ELISA (human serum samples)

Serum was isolated from whole blood samples using a serum separator tube and following clotting was centrifuged at 2000g for 10 minutes. Serum aliquots were stored at -80°C prior to analysis. Serum ACE2 was measured using enzyme-linked immunosorbent assay (HUFI00026 ^©^ 2018 Reagent Genie, Dublin, Ireland). An optimal serum sample dilution of 1:25 was determined. 100μl of standard solution and duplicate diluted serum samples were added to wells and incubated at 37°C for 90 mins. Wells were incubated with each of 100μl of biotin-detection antibody (incubated at 37°C for 60 minutes);100μl of HRP-streptavidin conjugate (incubated at 37°C for 30 minutes); 90μl of Tetramethylbenzidine substrate (covered and incubated at 37°C for10 minutes until emergence of blue color) with appropriate washing at each step. Finally 50μl of stop solution was added to each well and O.D absorbance was read at 450nm in a microplate reader (Thermoelectron™ multiskan ascent). The concentration of samples were determined using the extrapolated standard curve multiplied by the dilution factor.

### Statistical analysis

Categorical variables were described through absolute (n) and relative (%) frequencies. Continuous numerical variables were described as mean and standard deviation, or median and interquartile range (IQR), depending on distribution. Distribution of numerical data between independent groups was assessed with the Mann–Whitney U-test or independent Student’s t-test depending on the normality of the data. Distribution of categorical data between independent groups was assessed with a Chi-square test or Fisher’s exact test as appropriate. Spearman’s correlation coefficient (rho) was also used to assess the relationship correlation. Multivariate linear regression was used to evaluate serum ACE2 levels after normalization by log transformation. A p value <=0.05 was deemed to be significant. Statistical analysis was performed using SPSS V.25 and GraphPad Prism V 9.0.0.

## Results

### Animal model (mouse tissue)

**Figure 1:**
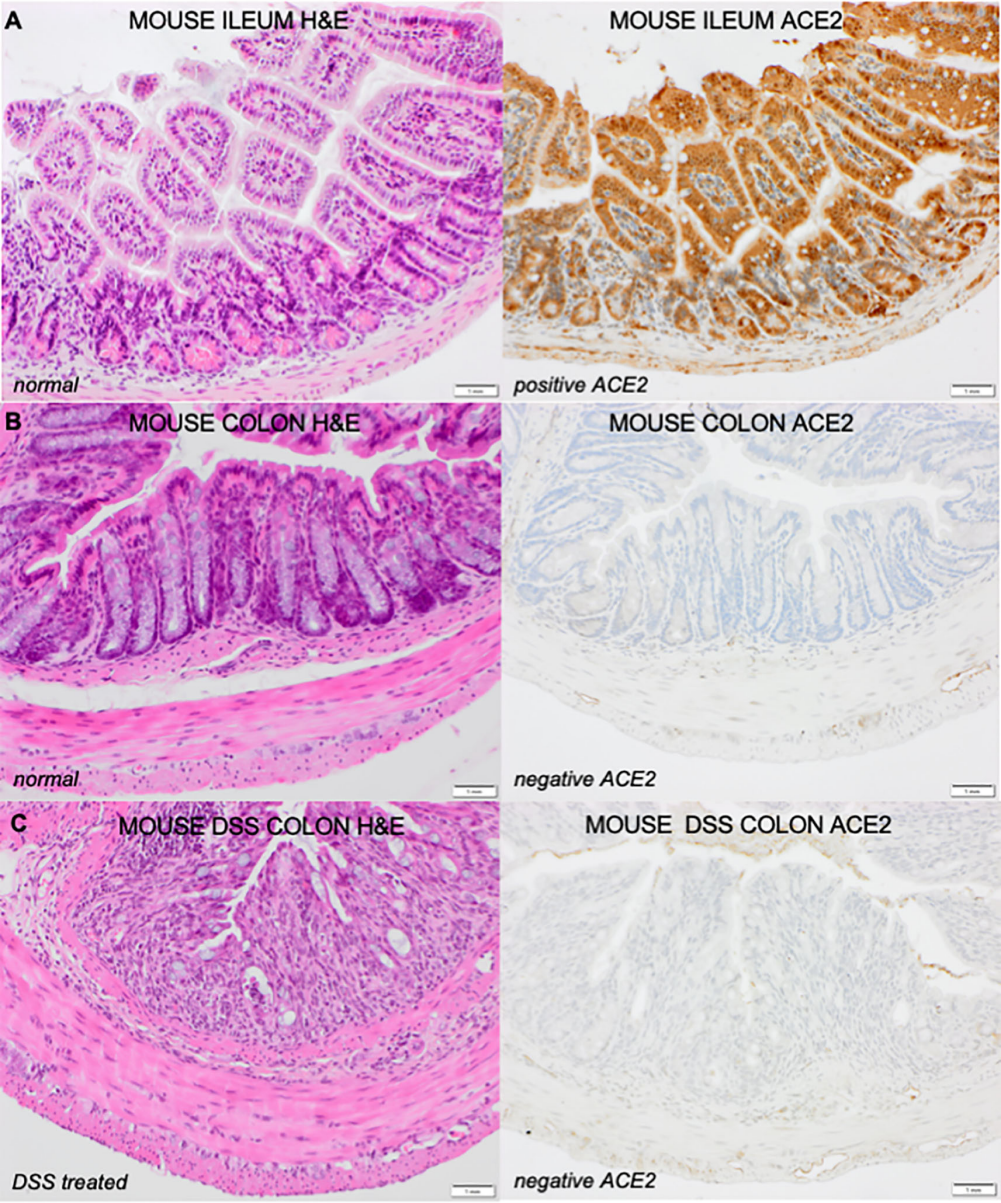
Corresponding H&E and ACEZ stained sections of **(A)** Healthy mouse lleum (Healthy mouse colon and **(C)** DSS mouse colon (n-5), magnification 20X The ileum and colon of healthy mice **(A, B)** and colon of Ds exposed mice **(C)** were stained with haematoxylin and eosin (H&E) for assessment of histological activity. H&E sections are represented on the left of the figure with the corresponding sections stained with ACE2 on the right.

ACE2 ileal and colonic tissue staining was assessed in healthy mouse ileum (Group A, n=5); healthy mouse colon (Group B, n=5); and in mice treated with DSS (Group C, n=5). Well characterized colitis-like symptoms were induced in female C57BL/6 mice in the DSS group. ACE2 staining patterns in the ileal and colonic sections are demonstrated in **Figure 1**. In healthy mouse ileum (Group A), ACE2 specific staining is localized to the luminal/brush border of intestinal epithelial cells. 80% of mice showed ileal ACE2 brush border staining with additional non-specific ACE2 staining localized to the basal and tip cytoplasm. ACE2 staining is predominately negative in mouse colonic epithelial cells, with no significant differences between the colon of healthy mice (Group B) and DSS exposed mice (Group C).

### Patient characteristics (human study)

**Figure 2:**
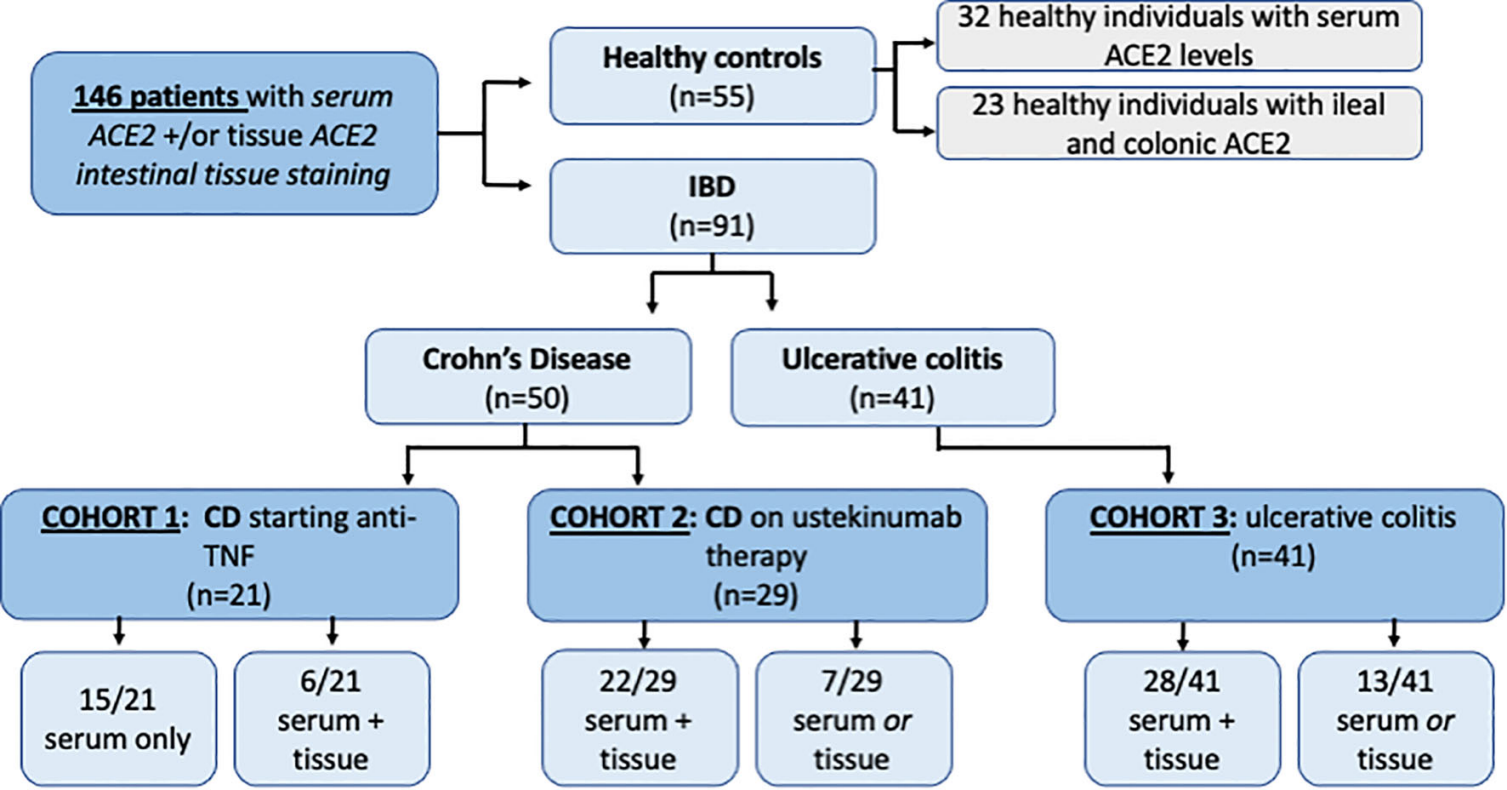
Inclusion flow diagram representing 180 patients and healthy controlsincluded inACEZ tissue and serum analysis.

The demographic and clinical characteristics of patients with IBD are presented in **Table 1**. A flow diagram summarizing the patients included is represented in **Figure 2**. A total of 146 subjects were included in the analysis. Of the 91 patients with IBD, 56 had contemporaneous serum and tissue samples for ACE2 analysis. Median age did not differ significantly between IBD (CD 36.5, IQR 27.5-48.5; UC 40.6, IQR 33.6-55.8) and control groups (34.0 IQR 27-48, p=0.218). There were significant differences in gender across groups with a higher proportion of female patients in the CD cohort and a higher proportion of male patient in the UC cohort (p=0.001), however overall gender balance was similar between IBD and non-IBD groups.

**Table 1:** Demographic and clinical characteristics of (A) all groups (Ulcerative Colitis, Crohn’s Disease and Healthy Control cohorts) and (B) patients with Inflammatory Bowel Disease.

(A)	Crohn’s Disease	Ulcerative Colitis	Healthy Controls	P value		
Age (y, Median; IQR)	37 (28-48)	39.9 (33.2-56)	34 (27-48)	p=0.218**		
Gender (Female, %)	40 (80%)	13 (31.7%)	26 (49.1%)	P=0.001*		
(B)	Crohn’s Disease (n=)	50	Ulcerative Colitis (n=)		41	P value
Disease duration (y) (Med, IQR)		9 (1-23)			8.9 (2.6- 14.7)	p=0.769**
Montreal classification (n)	*A1*	9 (18%)	*Disease extent*	*Proctitis*	4 (10.3%)	
	*A2*	32 (64%)		*Left sided*	15 (38.5%)	
	*A3*	9 (18%)		*Pancolitis*	20 (51.3%)	
	*L1*	8 (16.7%)				
	*L2*	20 (39.6%)				
	*L3*	21 (43.8%)				
	*Isolated perianal*	1 (2%)				
	*B1*	24 (48%)				
	*B2*	14 (28%)				
	*B3*	12 (24%)				
	*Perianal (yes)*	28 (56%)				
Disease activity	*Ileal Inflammation*	13 (26%)	*Mayo score*	*0*	5 (12.2%)	
	*Ileo-colonic inflammation*	7 (14%)				
	*Colonic inflammation*	6 (12%)		*1*	10 (24.4%)	
	*Active perianal disease*	3 (6%		*2*	15 (36.6%0	
	*Inactive/no evaluation*	21 (42%)		*3*	11 (26.8%)	
			*FCP*		58.5 (15-265.5)	
Medical therapy	*5ASA*				27 (65.9%)	
	*Immunomodulator*	17/42 (40.5%)			8 (19.5%)	
	*Biologic*	22 (44%)			7 (20.5%)	
	*Steroids*	3 (6%)			7 (17.1%)	
Smoking status (yes)		7 (14.%)			2/27 (7.4%)	p=0.359*
Previous resection		27 (54%)			4 (9.8%)	p=0.001*
**median test spss *Chi square						

### ACE2 immunohistochemical staining in the terminal ileum and colon of healthy controls (human tissue)

**Figure 3:**
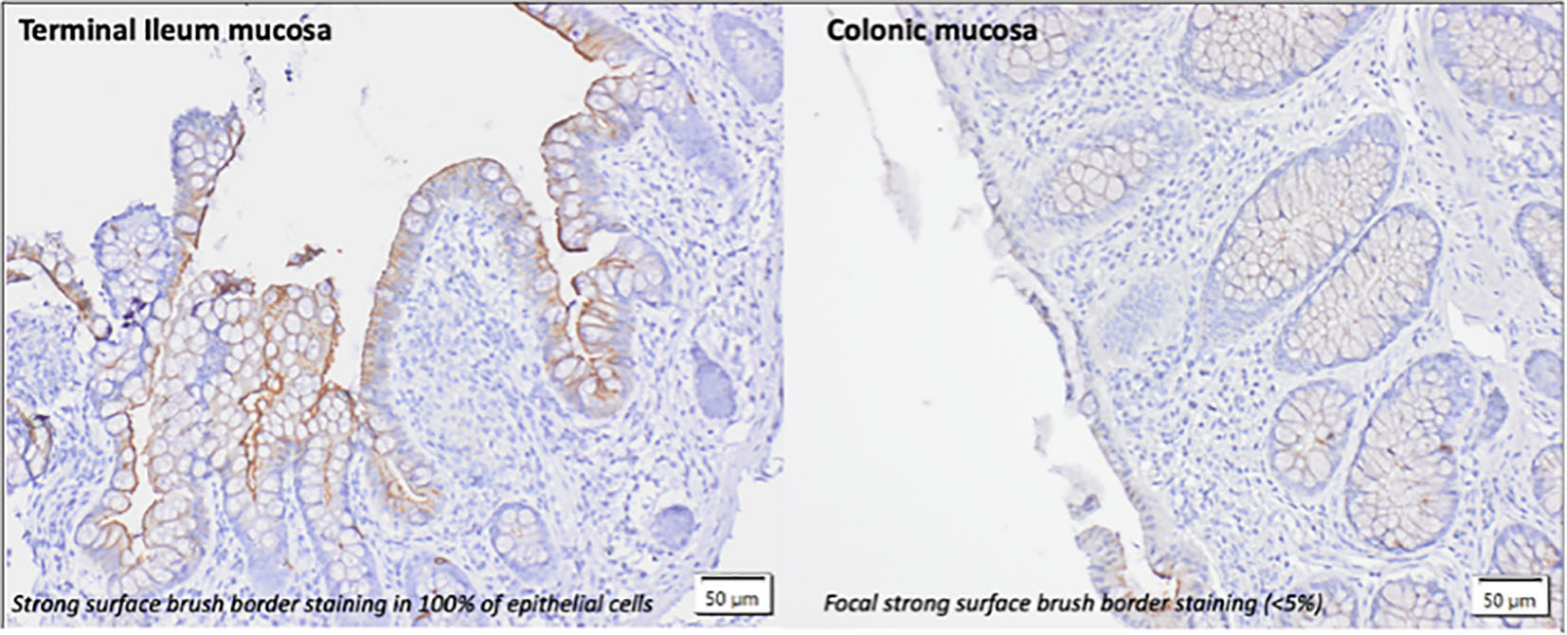
lmmunohistochemical pattern of ACE2 staining in the terminal ileum and colon of healthy controls without evidence of intestinalinflammation. Intestinal tissue biopsies (terminal ileum and colon) were stained with anti ACE2 Ab (1:100). The predominant form of staining (brush border)is demonstrated.

The ACE2 staining patterns in the ileal and colonic biopsies of healthy volunteers are demonstrated in **Figure 3**. ACE2 staining is predominately seen at the luminal/brush border of ileal and colonic intestinal epithelial cells. Intracellular (cytoplasmic) staining was not observed. In the terminal ileum of normal/healthy controls, >90% showed strong brush border staining (>80-100% of cells positive) with remaining controls showing less marked positive staining (<50% of cells positive). In the colon of normal/healthy controls, 87% (20/23 controls) were negative for brush border staining. A small number (8.7%) showed focal positive staining (<5% in 2/23 controls).

### Impact of inflammatory bowel disease on tissue ACE2 expression and relevance of disease activity (human tissue)

**Figure 4:**
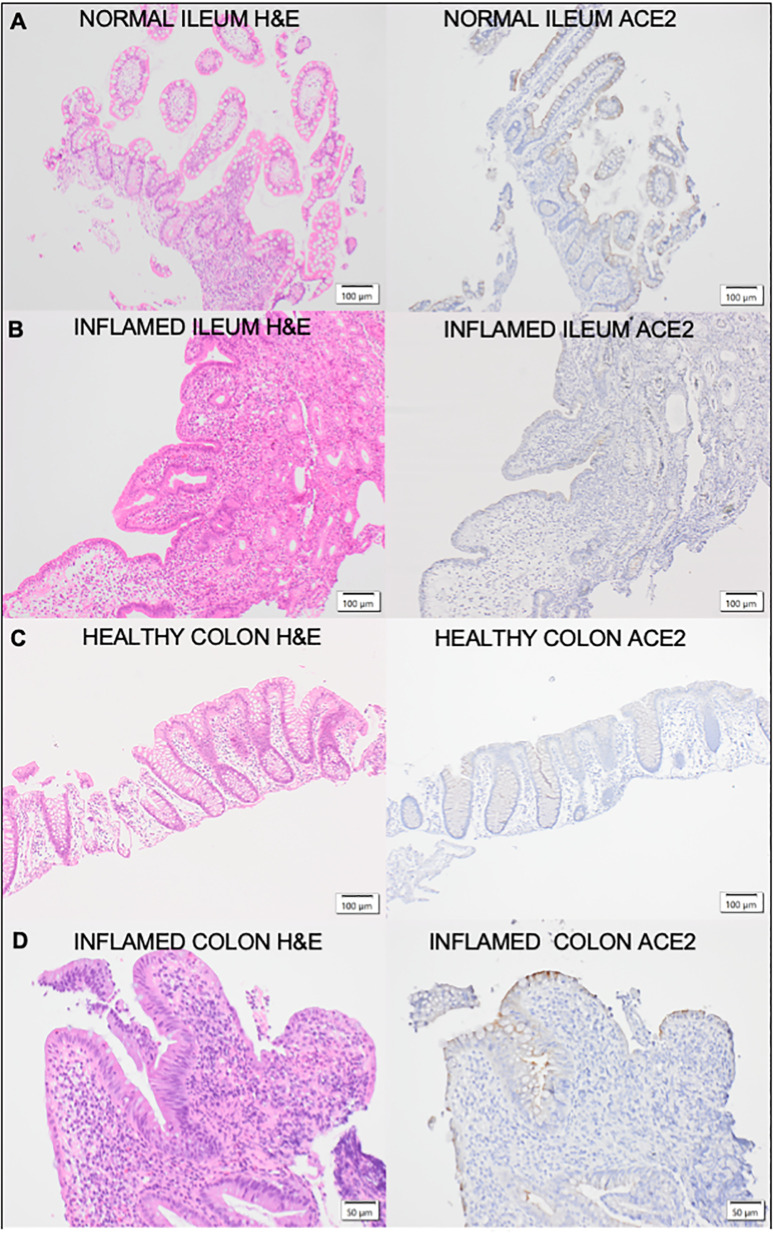
Corresponding H&E and ACE2 stained sections of **(A)** Healthy control ileaImucosa; **(B)** Inflamed Crohn's Disease ileal mucosa;**(C)** healthy control colonic mucosa and **(D)** Inflamed Ulcerative Colitis colon mucosa.The ileal and colonic mucosa of healthy controls and IBD patients were stained with haematoxylin and eosin (H&E) for assessment of histological activity. H&E sections are represented on the left of the figure with the corresponding sections stained with ACE2 on the right.

**Table 2** shows the levels of epithelial ACE2 observed in patients with IBD and demonstrates the differences observed in ACE2 tissue expression in active and inactive disease. Altered ACE2 staining patterns were identified in both the ileal (n=27) and colonic (n=84; CD=25, UC=59) epithelium of patients with inflammatory bowel disease compared to healthy controls. In the colonic epithelium from IBD patients, increased ACE2 staining was demonstrated compared to healthy controls (% staining, med. (IQR) 10% (0-40%) vs. 0% (0-0%)). In the ileal epithelium of CD patients, there was a reduction in ACE2 staining compared to healthy controls (% staining, med (IQR) 70% (40-100%) vs. 100% (80-100%)). Altered ACE2 staining was also identified in inflamed vs non-inflamed colonic and ileal biopsies in IBD patients. Median ACE2 staining (%) in inflamed colonic biopsies from IBD patients was 20% (IQR 5-45%) compared to 5% in non-inflamed biopsies (IQR 0-5%). In UC, colonic ACE2 staining was significantly higher in inflamed compared to non-inflamed biopsies (p=0.015). Similarly in CD, colonic ACE2 staining was higher in inflamed compared to non-inflamed biopsies, however this did not reach significance (p=0.393). In ileal biopsies, median ACE2 staining (%) was 80% (IQR 40-100%) in the presence of inflammation compared to 60% (IQR 45-100%) in non-inflamed biopsies. The ACE2 staining patterns and corresponding H&E staining in ileal and colonic biopsies from IBD patients are demonstrated in **Figure 4**.

**Table 2:** Changes in ACE2 intestinal expression in IBD patients and healthy controls in inflamed and non-inflamed colonic and terminal ileal biopsies.

		ACE2 intestinal expression (% staining)	
		Colonic epithelium med (IQR)	Terminal Ileum epithelium med (IQR)
Healthy controls		0 (0-0)	100 (80-100)
Crohn’s Disease	Non-inflamed biopsies	10 (0-20)	80 (40-100)
	Inflamed biopsies	40 (0-50)	60 (45-100)
Ulcerative colitis	Non-inflamed biopsies	5 (0-5)	
	Inflamed biopsies	20 (5-30)	

### Differences between serum soluble ACE2 in IBD patients compared to healthy controls (human serum samples)

Serum soluble ACE2 was found to be significantly higher in patients with Ulcerative Colitis compared to CD or healthy controls. Median serum ACE2 in patients with UC was 142.4 ng/mL (IQR 105.0-208.7) compared to 63.2 ng/mL (IQR 49.6-80.1) in CD and 91.8 ng/mL (IQR57.2-131.5) in healthy controls. This was corrected for disease activity at the time of sampling with active disease defined as an endoscopic Mayo score of ≥1 (UC cohort) or the presence of ulcers (CD cohort). In patients with UC, sACE2 level was found to be significantly associated with male gender (p=0.008) and disease activity (0.026). No association was identified between sACE2 and age (p=0.382), disease duration (p=0.636) or smoking status (p=0.513). In the CD cohort, there was no significant association between serum ACE2 levels with gender (p=0.815); age (p=0.235) smoking status (p=0.322); prior surgical resection (p=0.663); disease location (p=0.126); or behavior (p=0.416) as assessed by Montreal classification; presence of perianal disease (p=0.579) or disease activity (p=0.655). In healthy controls no association between sACE2 and gender (p=0.554) or age (p=0.670) was observed. Differences in serum ACE2 between groups are represented in **Figure 2**. Factors affecting serum soluble ACE2, and stepwise logistic regression are represented in **Table 3**. Of note, no significant correlations were identified between serum soluble ACE2 levels and ACE2 tissue staining in patients with inflammatory bowel disease.

**Table 3:** Demographic and disease related factors influencing soluble serum ACE2.

Variable		Number (n)	Serum ACE2 (Median, IQR)	Uncorrected p	Corrected p*
Healthy Control		29	91.8 (57.2-131.5)		
Crohn's Disease		44	63.3 (49.6-80.1)	0.001^a^	0.154
Ulcerative colitis		30	142.4 (105.1-208.7)		0.001
Age					0.084
Disease Duration					0.261
Gender	Male	55	121 (66.5-169)	0.001^b^	0.013
	Female	45	72.6 (53.4-92)		
Endoscopic disease activity	Inactive	10	88 (52.9-224.4)	0.749^b^	0.973
	Active	53	81 (57.8-135.3)		
Smoking status	Yes	8	79.3 (63.4-88.8)	0.830^b^	0.886
	No	64	75 (54.4-126.9)		
Steroid use	Yes	7	72.6 (54-82.7)	0.091^b^ **	0.012
	No	67	101 (66-150.8)		
Immunomodulator use	Yes	26	75 (54-148.7)	0.948^b^	0.073
	No	45	79.7 (57.8-125.6)		
Biologic use	Yes	24	57.3 (48.9-100.4)	0.008^b^	0.067
	No	48	81.5 (67.5-145.1)		
Previous resection	No	44	82.3 (67.5-145.8)	0.587^b^	0.786
	Yes	28	60.3 (53.3-98.1)		

Multivariate linear regression was used to evaluate serum ACE2 levels after normalisation by log transformation. A p value <=0.05 was deemed to be significant. Statistical analysis was performed wising SPSS V.25.

*serum ACE2 normalised by log transformation to perform stepwise backward linear multiple regression, p values corrected for age, gender, disease type, endoscopic disease activity, smoking status, immunomodulator/steroid/biologic use and prior resection.

**steroid use did not reach significance overall but was SS when applied to the UC cohort in both uncorrected and corrected analyses.

^a^Kruskal-Wallis, ^b^Mann-Whitney U.

### Differences between serum soluble ACE2 with treatment effect in IBD (human serum samples)

**Figure 5:**
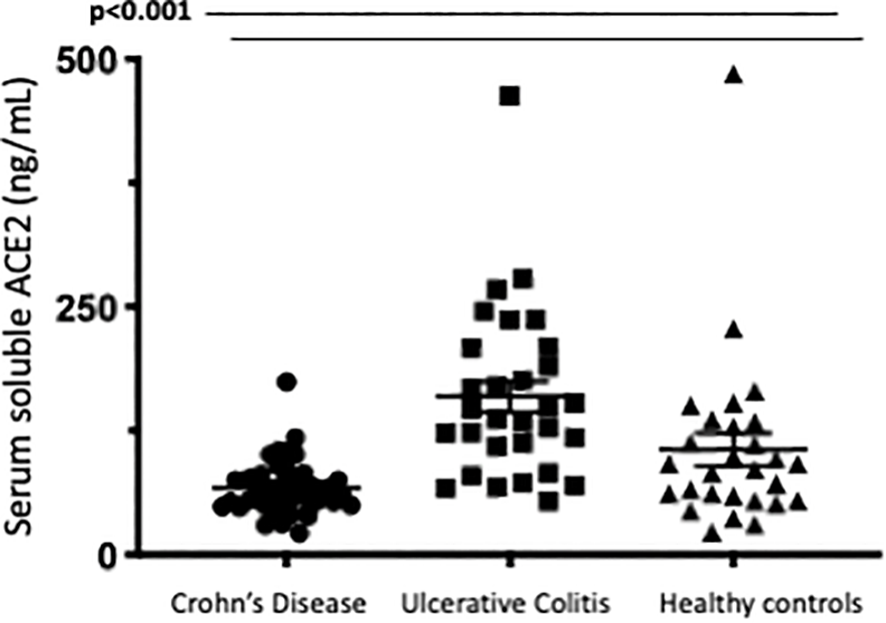
Serum soluble ACE2levels (ng/ml)in Crohns Disease, Ulcerative Col tis and Healthy control cohorts.Serum ACE2 levels were measured by ELISA.

**Figure 6:**
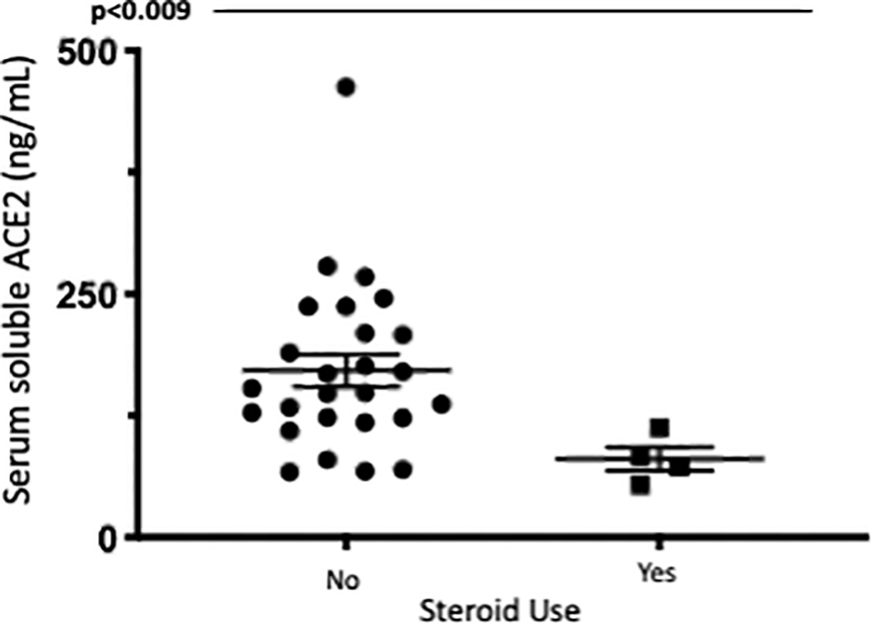
Serum soluble ACE2levels (ng/ml)in UC patients with and without corticosteroid exposure. Serum ACE2levels were measured by ELISA.

The most notable differences in sACE2 levels related to IBD treatment were significant differences in serum soluble ACE2 identified in patients with IBD on oral or IV corticosteroids, however, overall numbers were small limiting interpretation of this data. Median serum ACE2 was significantly lower in patients in the steroid group (p<0.012) especially in patients with ulcerative colitis (p<0.009). This remained significant when corrected for age, disease type and the presence of endoscopic disease activity. Additionally, median serum ACE2 was found to significantly reduce 14 weeks after anti-TNF administration in patients with Crohn’s Disease commencing anti-TNF. Patients in cohort 1 had serial measurements of serum ACE2 at baseline and 14 weeks after anti-TNF administration (72 (61.5-81.75) vs. 54.5 (39.5-72), p=0.026). Differences in serum ACE2 between healthy controls and IBD cohorts and between treatment groups are represented in **Figures 5** and **6**.

## Discussion

Significant gaps remain in our understanding of the role of the ACE/ACE2 pathways in the intestinal lumen. ACE2 is highly expressed on the luminal surface of intestinal epithelial cells where is stabilizes the neutral amino acid transporter B (0)ATI and functions as a co-receptor for nutrient uptake from food (14). ACE2 knockout mice develop severe colitis after intestinal injury, a phenomenon directly linked to deficiency of the amino acid, tryptophan and the resultant altered intestinal microbiome with down regulation of gut antimicrobial peptides (15). ACE2 in the gut may act as a regulator of the RAS, where it has anti-inflammatory and anti-fibrotic effects (16). One study on the effects of stress induced intestinal inflammation on the RAS, showed than the administration of the angiotensin receptor blocker, irbesartan, to mice, reduced colonic inflammation which correlated with increased ACE2 (17). GARG et al. found higher ACE2 staining in IBD compared to controls with similar tissue mRNA expression and higher plasma ACE2 activity in IBD compared to healthy controls (18, 19). Other groups using proteomic analysis of intestinal tissue found no alterations in ACE2 between controls and IBD but higher ACE2 expression in Crohns (20, 21). Finally, in another publication single cell RNA sequencing data was used to demonstrate that ACE2 expression in colonocytes was associated with genes regulating viral infection, innate and cellular immunity (22).

How the anti-inflammatory actions of ACE2 in the gut impact on the pathogenesis of IBD are yet to be elucidated. Here, we demonstrate clear differential ACE2 staining patterns in the ileum and colon of the healthy mouse and DSS mouse model; healthy human volunteers; and the changes that occur in IBD. Consistent with previous reports (23, 24), we demonstrate high levels of epithelial ACE2 staining in the ileum of the mouse model as well as healthy human volunteers with corresponding low levels in colonic epithelium. In addition, we show the alterations in ACE2 staining in intestinal inflammation in a murine model of acute inflammation (DSS mouse, day 5) and in the chronic inflammation that occurs in IBD. Interestingly in the DSS model, colonic inflammation did not lead to alterations in ACE2 staining. Our study is limited by the absence of a chronic murine model of intestinal inflammation, as well as known differences in the structure and expression of ACE2 between mice and human tissue (25, 26), however, we hypothesize that altered expression ACE2 expression profiles could be associated with chronic, rather than acute inflammation. Our findings confirm previously published data by Nowak et al (24) showing an increase in ACE2 tissue staining in the colon of IBD patients compared to healthy controls, where baseline levels are low and a reduction in ACE2 tissue staining in the ileum of CD compared to healthy controls (11, 27). We demonstrate that inflammation appears to modulate these changes with higher levels of ACE2 staining in the inflamed colon of IBD patients and reduced ACE2 in the inflamed ileum.

There is a paucity of data relating to sACE2 levels in IBD. Here we provide evidence of differential serum sACE2 profiles in IBD patients and the alterations that occur with medical therapies and disease activity. We identified significantly higher levels of serum sACE2 levels in patients with Ulcerative Colitis compared to Crohn’s Disease and healthy controls. Patients on steroid therapy had reduced levels compared to those not on steroids, particularly in those with ulcerative colitis. Additionally, those on biologic medications had lower sACE2 levels and biologic therapy led to a reduction in sACE2 levels in a cohort of CD patients with serial ACE2 measurements. Few studies have evaluated sACE2 levels in patients with IBD, particularly in the serum. Garg et al. found higher overall levels of plasma ACE2 in IBD but didn’t note any changes with IBD therapies (18). The causes of differential ACE2 serum profiles in inflammatory bowel disease are unclear but may relate to excessive shedding due to upregulation of the TNFα convertase, ADAM-17 in inflammation (6). Additionally, they may imply a compensatory ACE2 response to inflammation, as part of the counter-regulatory arm of the RAS (16). The recent interest in recombinant human ACE2 as a possible therapy for SARS-CoV2 (28) raises the importance of understanding the role of sACE2, and in particular the impact of medication use that may affect viral susceptibility.

Finally, our data found no correlation between circulating levels and tissue expression of ACE2 in patients with IBD. The recognition of a local intra-organ RAS (29) under paracrine and autocrine control in the gut and other organ systems supports this finding with increased cleavage of membrane bound ACE2 circulating in the blood under inflammatory conditions.

Several limitations to the study must be acknowledged. The functional significance of altered ACE2 expression in the intestine remains unclear and any potential association with the risk of SARS-COV2 viral inclusion are hypothetical. Furthermore, limited numbers precluded further stratification of tissue and serum ACE2 according to disease location or behaviors. In addition, limited numbers of patients analyzed in the biologic or steroid treatment groups, means these results should be approached with caution and further data is required to support this signal.

In conclusion, our findings provide an improved understanding and strengthen the existing knowledge base on alterations in serum and tissue ACE2 expression in patients with IBD. Further research to better elucidate the pathways responsible for the alterations of ACE2 in the gut is required, and in particular how they relate to the pathogenesis of IBD.

## Data Availability

The raw data supporting the conclusions of this article will be made available by the authors, without undue reservation.
